# Block or Cylinder Filling

**Published:** 1857-06

**Authors:** James Taylor


### BLOCK OR CYLINDER FILLING.

BY JAMES TAYLOR.

Time was when the operation of filling teeth held not that pre-eminent place in the profession which it now does. Many of our old practitioners can well recollect the time when the insertion of pivot teeth was regarded as one of the most important operations, if not the most frequent, of Dental Surgery. From 1827 to 1833-35, the insertion of pivot teeth was generally resorted to far more frequently than since that time. In lately looking over memorandum books kept during that period, I have been struck with the relative proportion of pivot and plate teeth then inserted. Take the present time, and this proportion is fully reversed. The number of fillings then put in did not begin to compare with the present ratio in any given amount of practice.

I presume that gentlemen at that time in full practice in our Eastern cities had a larger amount of filling to do in proportion to their other practice than was customary with our Western operators.

In the West, at that time, the larger part of the community regarded dental operations more ornamental than useful—hence many would have teeth inserted without any thought of preserving others by filling. Teeth were then cut off for pivoting which would now be regarded as well worth filling; and which, indeed, it might be regarded mal-practice now to do anything else but fill.

The view which I have here presented, I think, will be fully sustained by the published works of the profession. In looking over most of our old Dental books, (and which were the only ones the profession had access to twenty-five years ago,) although all recommend the operation of filling as of importance, yet they give but little positive instruction as it regards the manipulation. Take, for instance, Koecker, the standard work of that time, and indeed the best work we had up to that time, and although much is said in relation to the operation which is right and proper, and which would be of interest to the general reader or the public, yet a small paragraph embraces all he says of the actual manipulation.

"In performing," he remarks, "the second part of the operation, the stopping of the tooth, the metal should be firmly pressed into the cavity, and rendered as compact as if it were solid metal, so that nothing could by possibility penetrate through it. The operation is then to be completed in the following manner: The redundant metal is to be cut away, and the plug perfectly smoothed and polished by some burnishing instrument."

Now this description is enough for the patient; but what is there by which the student could be guided? In speaking of the "Mechanical implements for stopping teeth," he remarks that "I find, for this operation alone, more than one hundred and seventy separate implements ready for use," but not one of these is actually described.

In looking over other authors up to this time, and even later, we find but little that is more explicit. It is true Desirabode says something about the manner of preparing the gold for its insertion into the cavity, and, that after a blunt pointed instrument has been used for forcing it in, a sharper pointed one should be used to consolidate the edges; yet but little is said by which the student could be guided in the operation. There is, indeed, nothing in all of our earlier works which will at all compare with that minute and explicit description which we find for other operations of general surgery. I have been tempted to run out a parallel of such cases, and bring up this one operation of Dental Surgery to the present time; but I find that it would occupy more time than I have at present to spare, and would also take too much space for one article in the Register. These remarks, however, appear to be necessary as introductory to what I have to say on "Block or Cylinder Filling." We have got beyond that point in the history of our profession where it is necessary to argue its importance, and I am glad to see that the profession is more interested to know which is the best method of operating.

I do not suppose it possible that acquired modes of filling teeth, the manipulation of which has been mastered by old operators, should be suddenly thrown aside for new ones. Indeed it would not be best to do so, yet a mode which has peculiar advantages should be tried, under advantageous circumstances, until it is also mastered, and then it will certainly be generally practiced. It is only in this way that perfection can be attained.

I am satisfied that gold or tin foil rolled up into blocks or cylinders to meet the varied cavities, can be introduced with proper instruments, in much less time, with far more accuracy, and so as to make a superior filling than in any other way. The advantages, then, of this mode is that more of the labor is done out of the mouth than in any other way. The position of the gold in the cavity, with one end of each lamina at the bottom of the cavity and the other protruding from its mouth, gives an adaptation of gold in the best possible manner for its compactness, and finally for a good finish to the plug.

But, say some, it is impracticable, except in favorable cavities. I might remark that every cavity should be made favorable. No mode gives certain success, unless the cavity is readily approached. In those which, from their position, are the most difficult of access, there is nothing which will so effectually aid in the success of the operation as to get in one or more blocks, properly adjusted, as a foundation for the balance of the gold to be introduced upon. I have found in cavities the most difficult of access, that a block of gold rolled up as hard and compact as possible, and as large as can be with certainty introduced into the cavity, facilitates the operation more than any thing else. If such cavities are properly formed, and this block properly adjusted in the same, the remainder of the operation is easily accomplished, and we have the best possible assurance that the plug is a good one. Take, for instance, a cavity between the molar teeth, either upper or lower, the space at the cutting edges of the teeth is made as wide as the cavity to be plugged is deep, and this is a good rule, although not absolutely necessary for the introduction of the block of gold. The upper wall of this cavity having been formed a little enlarged as it passes in, the first block of gold is forced against this. If now this block is forced into the bottom of the cavity, and is sufficiently large, it may be so compressed before any other gold is introduced, that the most difficult and important part of the plug is perfect, without any doubt. The remainder, being the most accessible, will give but little trouble.

I cannot conceive of any other mode of introducing the gold so simple and so certain, and, at the same time, accomplishing the object we all aim at. It will be seen, however, that this conflicts somewhat with the directions some give for filling with cylinders, which is to place the cylinders first around against the walls of the cavity, and finish in the centre. I hold that no general direction of this kind is generally admissible. The location of the cavity, its general form, the strength of its walls, &c., must govern us in the adjustment of the blocks of gold. The only general rule is, when the walls of the cavity are strong, to commence at the farthest wall and end at the anterior—say in approximate cavities, commence at the part nearest the gum; in the central cavities of the molars, at the posterior part of the tooth, &c. After the blocks have all been introduced, and, as introduced, forced compactly to these places, a sharp pointed plugger should be passed over the whole surface of the plug, and if a soft place is found, the plugger is to be forced in, and an opening as large as possible made, which must then be filled. I have always on hand a few small cone like blocks rolled very hard, and at once select one which can be forced into such a place, and which will completely fill up the cavity. In large fillings we may have two or three such to put in at different places. This, however, will depend very much on the general care with which the blocks have at first been introduced.

Experience has taught me that the block should be very little longer than the cavity is deep—the superfluity in length of blocks, (if properly introduced and consolidated as introduced) is so much waste gold, for it must all be filed away. The laminæ of gold when closely compressed as they should be, will not permit any breaking down by pressure on the ends of the blocks.

In this mode of filling, care should be taken to have the cavity properly prepared, and particularly that the cavity should not be much "nicked," and when any enlargement does take place, the wall of the cavity when enlarged should be as near straight as possible, so as not to require the bending or doubling up of a block to fit it. Any one practicing this mode will soon find how easy it is to fill some cavities which heretofore he may have regarded as difficult. If, for instance, two portions of the walls of a cavity somewhat antagonistic can be properly formed to receive each a block, the remainder of the cavity may be well filled with cone shaped blocks, the point of the cones forced between the two first blocks. A little tact in this way will enable the operator to round out and fill up a cavity which, in any other mode, must be done on the same principle required in the use of crystal gold, and where even the moisture of the patients breath is an obstacle to success.

It will be seen from the above that some tact is necessary in making the blocks, so that we may meet these emergencies.

In my first manufacture of these blocks, they were all, or nearly all, made by being rolled on the point of a straight pair of plugging pliers. In some cases I still use an instrument of this kind to give a particular form to my block; but the method of forming these blocks on a broach, as recommended by Dr. Clark, of New Orleans, is much more speedy, and, as a general thing, we now thus make our blocks.

In closing this article on Block or Cylinder filling, I wish to allude to a few facts in relation to the introduction of this mode of filling teeth, which some in the profession do not appear to understand. In the introduction of any thing new in practice, and particularly if there are some excellencies about it, many instantly lay claim to priority. In relation to that now under consideration, there appears to be a singular, and rather ludicrous misconception of the whole affair. At the Meeting of the "Missippi Valley Association of Dental Surgeons," in 1849, the subject of filling teeth was up for discussion; and after some considerable talk on the mode of filling, I was requested to prepare an address on the subject for the next meeting. This was in part accomplished, but as the subject appeared to require more time and space than could be thrown into one article, I was requested to continue the subject and publish in the Register. This accounts for the series of articles on this subject, commencing in the October number of the Register for 1850. In my description of this mode of filling I used the appropriate, but not very euphonious or classic name of "blocks." This very unharmonious blunder in a name has evidently misled many who have been taken with the name of cylinders, taking it for granted that the new name was a new mode also. Many years ago it was a very common method of filling teeth with gold rolled into cylinders, but this was a roll nearly as long as the sheet of foil, and was introduced very much as was the strip which succeeded it. This accounts somewhat for the rejection of the name in my article. Some twenty years ago, much was said about balls or pellets, which were introduced on the end of a plugging instrument, and packed into the cavity much as crystal gold is now used. The idea, however, of getting the gold into the cavity in regular laminated order, substituting somewhat nature's order in the enamel itself, I believe, had never been recommended to the profession before the articles I published on block filling.

I do not say that others were not filling teeth in this manner perhaps long before I was; yet, unfortunately, the great idea of secrecy kept the profession generally in utter ignorance of the fact. I must confess, however, that I cannot still see how any one can use blocks aright, and to advantage, without the plugging forceps which I introduced. Without these, I should certainly fall back on the strips.

I know not, therefore, whether Dr. Badger, Dr. Cox, Dr. Laird, or Dr. Clark, had been filling teeth in this manner before the appearance of my articles. Dr. Clark, however, I believe, does not make such claim; yet his remarks, as reported at Chicago, might thus be interpreted, for he talks of having "established my (his) present mode of cylinder filling," and then remarks that, "after a year or two found out that Dr. Badger did not fill with cylinders."

In relation to Dr. Laird, I can only state that after I had written my articles, I received a letter from him stating that I had taken his "thunder," and that my block filling was what he had been practicing as a secret. I knew, before this, that Dr. Laird claimed a superior mode of filling teeth, which he saw proper not to disclose. I state this in justice to those who have used his name in this connection.

The Dr. soon saw from my articles on block fillings that his cylinders were my blocks, Dr. Clark, I believe, only claims an improved method of making these blocks; and, for this method, the profession should, as far as I know, give him credit.

In conclusion, I would say that it is a matter of no little importance to the profession to know that mode of introducing gold into the different cavities to be filled, which will accomplish it in the best manner and quickest time. Let us say what we may about always keeping cavities and the gold dry, yet the fact is indisputable, that many of the most difficult cavities to fill will weary the patient and hurry the operator if he gets the gold introduced before any moisture gets in the way, even if he selects the most expeditious method.

I had determined, sometime back, to say nothing about the introduction of the method of filling teeth with blocks or cylinders, thinking that a careful review of our periodicals would put the profession right on the subject—but many forget what was written six or seven years ago, and others scarce go back so far in their reading, while others have been asking again and again for some explanation of this matter.

The publication of a few letters received in 1850 and 1851, during the time the articles on block filling appeared in the Register, would, if not out of place, give the views of many on this subject, but, for the present, *quantum sufficet.*

